# Guidelines for Complicated Urinary Tract Infections in Children: A Review by the European Society for Pediatric Infectious Diseases

**DOI:** 10.1097/INF.0000000000004790

**Published:** 2025-03-19

**Authors:** Penelope A. Bryant, Maria Bitsori, Kalliopi Vardaki, Nina Vaezipour, Maria Khan, Michael Buettcher

**Affiliations:** From the * Departments of Infectious Diseases and Hospital-in-the-Home, Royal Children’s Hospital, Melbourne, Australia; † Clinical Infections, Murdoch Children’s Research Institute, Melbourne, Australia; ‡ Department of Paediatrics, University of Melbourne, Melbourne, Australia; § Department of Paediatrics, Heraklion University Hospital, Heraklion, Greece; ¶ Department of Nephrology, Great Ormond Street Hospital for Children, London, United Kingdom; ‖ Departments of Paediatrics and Nephrology, University of Crete, Heraklion, Greece; ** Department of Pediatric Infectious Diseases and Vaccinology, University Children’s Hospital Basel, Basel, Switzerland; †† Mycobacterial and Migrant Health Research Group, University of Basel and Department of Clinical Research, Basel, Switzerland; ‡‡ Department of Microbiology, Pathology Laboratory, Peshawar Institute of Cardiology-MTI, Peshawar, Pakistan; §§ University of Basel, Basel, Switzerland; ¶¶ Pediatric Infectious Diseases, Department of Pediatrics, Children’s Hospital of Central Switzerland, Lucerne, Switzerland; ‖‖ Pediatric Pharmacology and Pharmacomentrics Research Center at University Children’s Hospital Basel (UKBB), Basel, Switzerland; *** Faculty of Health Sciences and Medicine, University Lucerne, Lucerne, Switzerland.

**Keywords:** urinary, infection, complicated, pediatric, neonatal, urological

## Abstract

**Background:**

Complicated urinary tract infections (cUTI) present a challenge to the clinician because of the variety in clinical syndromes included and consequent difficulties in synthesizing evidence. A harmonized definition of cUTI does not exist. In national guidelines, management recommendations for cUTI are often neglected. We aimed to define the four most important controversies and formulate management recommendations for cUTI in children and adolescents.

**Methods:**

The European Society of Pediatric Infectious Diseases Guideline Committee convened a working group of experts from microbiology, pediatric nephrology and infectious diseases with expertise in managing children with UTI. A comprehensive literature review was done using PubMed, Embase and the Cochrane library to find studies in children under 18 years published until December 2024. Four controversies were defined from experience and available evidence. Children with cUTI were categorized into 5 subgroups: anatomical/functional urological abnormalities, multiple UTI recurrences, severe clinical presentation, nonurological underlying conditions and neonates. Respective management guidelines were formulated through the evidence and by consensus of working group members. Recommendations were made using GRADE criteria.

**Results:**

The term cUTI is generally used to define children with UTI with an increased likelihood of failing conventional management. The included 5 subgroups are the most likely to need additional investigations at diagnosis and during the course of infection, initial intravenous antibiotics, longer treatment duration, antibiotic prophylaxis, follow-up imaging and surgical referral. These are detailed for each subgroup.

**Conclusions:**

These comprehensive guidelines offer evidence-graded recommendations specifically for pediatric cUTI, addressing gaps that exist in current guidelines.

## INTRODUCTION

Complicated urinary tract infections (cUTI) present a challenge to the clinician because of the variety of clinical syndromes included and consequent difficulties in synthesizing evidence for practical guidance. The cumulative incidence of all childhood UTI by the age of 7 years is 1%–2% in boys and 7% in girls.^1,2^ Of those presenting to emergency, 5%–24% are deemed cUTI, so it is a common childhood infection.^2,3^ However, defining what makes a UTI complicated is difficult because it has been characterized by a variety of UTI syndromes, which include hosts with abnormal urology or other underlying conditions, severe clinical presentations with or without extensive tissue involvement, and unusual pathogens. For this review and guideline, cUTIs are those that require other management considerations than straightforward lower or upper tract UTIs for which evidence and guidelines exist. Accurate diagnosis and management of cUTI limit morbidity and mortality from sepsis and long-term renal damage. However, most trials of UTI in children have excluded those with cUTI, and consequently, many guidelines also exclude them. The absence of a standardized definition for cUTI has resulted in inconsistencies in treatment approaches, which may lead to increased morbidity among affected children. Existing major UTI guidelines for children either do not address complicated UTIs at all or are limited to only some complications or some age groups.^4–6^

This review aims to address the four most important controversies when managing children with cUTI and provide recommendations for definition, investigations, treatment and follow-up. The resulting guidelines offer evidence-graded recommendations specifically for the management of pediatric cUTI, addressing gaps that exist in current guidelines.

## METHODS

The European Society of Pediatric Infectious Diseases Guidelines Committee convened a working group of experts from microbiology, pediatric nephrology and infectious diseases. The working group conducted a comprehensive literature review using PubMed, Embase and the Cochrane library of studies published until December 2024. Search terms included: urinary tract infection/UTI, complicated, complex, atypical, urinary tract abnormality/anomaly, recurrent/recurrence, chronic renal/kidney disease/failure, immunosuppression, adolescent, child, pediatric, infant, neonate/neonatal, definition, investigations, diagnosis, treatment, management, antibiotics, prophylaxis and imaging. Studies were included for review if they involved children under 18 years with any definition of cUTI and excluded if they focused solely on uncomplicated UTI (uUTI) or adults. Types of included studies were reviews, trials, comparative and cohort studies and existing guidelines were also reviewed for references. Four key controversies were identified from experience and available literature: definition of cUTI, investigations, treatment and follow-up. Evaluation of evidence quality and formulation of recommendations used GRADE methodology.^7^ After assessment of the literature on definition, cUTIs were categorized into 5 main subgroups, and respective management guidelines were formulated from the graded evidence by working group consensus.

## RESULTS

### What Is the Definition of cUTI?

- cUTI is most usefully defined as children with UTI with an increased likelihood of failing conventional management (GRADE D).
- Children with cUTI have one or more of:
  - localized risk: significant anatomical or functional urological abnormality, multiple recurrent UTI,
  - generalized risk: neonates, nonurological underlying condition (renal and nonrenal) and
  - severe clinical presentations: sepsis, severe renal parenchymal disease.
- We have used “complicated” to define subgroups at presentation and consequent management recommendations and “atypical” to refer to unexpected clinical progress (GRADE D).

The term “complicated UTI” has been used to describe multiple different UTI syndromes, with additional characteristics that might complicate the usual UTI course, variably including severe clinical presentations with or without extensive tissue involvement, hosts with abnormal urology or other underlying conditions and unusual pathogen properties.^8,9^ This variety makes it difficult to compare studies and provide consistent evidence in guidelines for the management of cUTI. UTI has traditionally been categorized as uncomplicated (uUTI) and complicated (cUTI) to standardize trial participants, but due to the lack of specific and clearly defined criteria at the outset, the definition has become increasingly broad and ambiguous. In a recent systematic review in adults, cUTI was variably defined by host factors and systemic involvement, a combination of both or neither.^10^ In children, early attempts to define cUTI variably included all neonates, most children with pyelonephritis, children with known anatomical or functional abnormalities, extensive parenchymal involvement, septicemia, nosocomial acquisition, renal impairment and recent antibiotics.^9,11^ The limited number of pediatric studies addressing cUTI (Table S1, Supplemental Digital Content 1a, http://links.lww.com/INF/G139 and 1b, http://links.lww.com/INF/G140), have used various definitions, from comprehensive to brief to single complication.^3,12–26^ Regarding the cUTI definition in existing guidelines for childhood UTI management, the updated National Institute for Clinical Excellence guideline for childhood UTI^27^ and recent Canadian^28^ and Swiss^29^ guidelines used varying combinations of severe presentation, renal issues and failure to improve in their definitions, while the Italian Society of Pediatric Nephrology^30^ uses severe presentation as the only criterion.

The most systematic effort to categorize the diversity of UTI syndromes and provide a basis for the definition of cUTI has been by the European Association of Urology (EAU), through a four-component classification system that identifies children likely to fail conventional UTI management.^5^ This incorporates clinical presentation, severity of tissue involvement, host risk factors (ORENUC acronym, O: no risk factors, R: recurrences, E: extra-urological risk factors, N: nephropathic diseases, U: urological risk factors, C: catheter) and pathogen susceptibility. Most of the broadly recognized subgroups of cUTI include components of the EAU system and are incorporated into the 2015 EAU/ESPU Guideline,^31^ although the 2021 revision does not include neonates.^5^ Uncomplicated pyelonephritis is no longer considered cUTI. Part of the problem with clearly defining cUTI is that the spectrum of disease is wide, with multiple mild to severe complicating factors. It has been found that if more complicating features exist, clinicians are more likely to manage these differently from uUTI, that is, use intravenous (IV) antibiotics.^32^ It may be that rather than categorizing UTI as either uncomplicated or complicated, the concept of less complicated or more complicated is useful, with an increasing number of abnormal features guiding more aggressive management. This requires further study.

Despite the broadness and inconsistencies in the term cUTI, it is preserved in the present guideline, where we use “complicated” to refer to components at presentation that impact immediate management and “atypical” to refer to progress that does not follow the expected trajectory. We define several subgroups of children with UTI that are deemed complicated at presentation to differentiate them from children with uUTI and to consider their diagnosis and management separately, rather than addressing them as a heterogeneous whole. This has been done considering published literature, other UTI guidelines and the EAU classification system. All subgroups identified in this guideline have at least 2 components from the EAU classification system^5^ (Table 1).

**TABLE 1.** Subgroups of cUTI Defined in the Guideline

cUTI Subgroup	Reason for Inclusion (Number of Components Aligned With EAU Classification System)	Evidence Grade
Known significant anatomical or functional urological abnormality (including post instrumentation)*	High risk for infection complications (nephronia and abscess) and high risk of recurrences or unusual pathogens especially when in prophylaxis (3)	D
Multiple recurrent UTI†	High risk of unusual pathogens or undiagnosed significant urological disorder (2)	D
Severe clinical presentation‡	High risk for impaired renal function and extensive tissue involvement and systematic involvement (2)	D
Nonurological underlying conditions	High risk for severe presentation, extensive tissue involvement (nephronia, abscess and emphysematous pyelonephritis) and unusual pathogens (3)	D
Neonates	Immature immune system, high risk of bacteremia and high risk of unusual pathogens (3)	D

* Not grades I and II, nondilating VUR or mild antenatal hydronephrosis.

† Not asymptomatic bacteriuria.

‡ Not fever alone.

### Which Investigations Are Needed at Diagnosis and During Complicated UTI?

- Patient history and clinical review underpin the diagnosis of UTI and assessment of complicating features at presentation (GRADE D).
- Urine collection should be done by clean catch, catheterization or suprapubic aspiration and urine analysis, culture and susceptibility testing should be undertaken for all children (GRADE C).
- Additional blood investigations for cUTI may include inflammatory markers, full blood count, blood culture, urea, creatinine and electrolytes (GRADE C).
- An acute ultrasound scan is helpful in some clinical situations (GRADE D).
- All children with cUTI should be reassessed for treatment response at 48 hours. If progress is atypical, consider complications and/or differential diagnosis (GRADE D).
- Repeat urine testing is only needed if there is inadequate treatment response (GRADE D).

All children, irrespective of uUTI or cUTI, need urinary investigations to confirm UTI diagnosis^33–37^ (Table 2). Key investigations and their interpretation are given below.

**TABLE 2.** Summary of Diagnostic and Other Initial Investigations in Children With cUTI

Diagnostic Investigations for All Children With Suspected UTI			Evidence Grade
Urinalysis	Useful for all children suspected for UTI False (+): postsurgery/instrumentation, inflammatory conditions, eg, Kawasaki disease False (−): neonates, pathogens with low inflammatory response, eg, *Enterococcus* spp., neutropenia		C
Urine culture	Gold standard for UTI diagnosis False (+): contamination, children with functional conditions False (−): prior antibiotics, some severe parenchymal disease (nephronia and abscess)		C
Additional investigations for children with suspected/confirmed cUTI			
cUTI subgroups	Differences from uUTI	Investigations to consider	
Known significant urological abnormality*	High risk for infection complications (acute lobar nephronia/acute focal bacterial nephritis and abscess) and high risk of recurrence or unusual pathogens especially when on prophylaxis	Inflammatory markers, urea and creatinine USS should be performed early to evaluate for the presence of a kidney abscess	B–D
Multiple recurrent UTI	High risk of unusual or resistant pathogens or undiagnosed significant urological disorder	Inflammatory markers, early USS to evaluate for renal abscess USS if not done previously to detect urological abnormalities	A–D
Severe clinical presentation	Toxic presentation, impaired renal function and extensive renal tissue involvement	Blood cultures, full blood count, renal function tests, serum electrolytes and USS	A
Nonurological underlying conditions	Immunocompromised, diabetes, high risk for severe presentation, extensive tissue involvement (nephronia and abscess) and unusual pathogens, eg, fungus	Inflammatory markers USS should be considered early to evaluate for the presence of a kidney abscess	B-D
Neonates	Immature immune system and high risk of bacteremia (group B streptococcus and *E. coli*)	Blood/CSF cultures, full blood count, inflammatory markers, serum electrolytes, renal function tests and USS	B-D

CSF indicates cerebrospinal fluid.

* Including postsurgery/instrumentation.

As with uncomplicated UTI, clean catch urine collection is preferred due to its noninvasive nature, with catheterization or suprapubic aspiration being the gold standards if collection is difficult or diagnosis is uncertain. In infants under 2 months, clean catch methods are challenging and can result in up to 38% contaminated samples, so catheterization or suprapubic aspiration is advised.^38^ Up to 88% of specimens collected via a urine bag with a positive culture will be false positives, so they should only be used in nonurgent situations as an initial screening measure; a negative result confirms the absence of a UTI.^39^

### Urine Dipstick Analysis

If leukocyte esterase is greater than trace, pyuria is present. Dipsticks may be false negatives in children with <3 months with high voiding frequency or with *Enterococcus* spp. or *Pseudomonas aeruginosa*.^40^ These pathogens have a reduced ability to convert nitrate to nitrite, and *Enterococcus* spp. may not induce a strong inflammatory response to increase leukocyte esterase.^37,40–42^ Dipsticks may be false positives due to contamination, fever of a different cause, localized or systemic inflammatory processes, for example, Kawasaki disease.^28,43^

### Urine Microscopy

Pyuria is present with ≥5 WBC/high-powered field with standardized or automated microscopy or ≥10 WBC/mm^3^ on a hemocytometer. Because of low sensitivity, negative urine microscopy does not exclude UTI.^44^

### Urine Culture

There are differing definitions of positive urine culture. The American Academy of Pediatrics defines the quantity of bacteria for a UTI as >50,000 colony-forming units/mL.^39^ In young infants (≤90 days) with frequent urination, 1000–10,000 CFU/mL in catheter specimens may indicate UTI. Clinically relevant uropathogens include *Escherichia coli*, *Klebsiella* spp., *Proteus* spp., *Enterobacter* spp., *Citrobacter* spp., *P. aeruginosa*, *Serratia marcescens*, *Enterococcus* spp., *Staphylococcus saprophyticus* and *Streptococcus agalactiae.*^28^ In general, growth of ≥2 different bacterial species suggests contamination.^45^ *Lactobacillus* spp., coagulase-negative staphylococci (CoNS) and *Corynebacterium* spp. are generally not relevant, including in the context of indwelling catheters. However, CoNS may be relevant in immunocompromised children or preterm infants (see below).

Urine culture alone should not be used to diagnose UTI (bacterial presence may just reflect bacteriuria) but should always be considered in the context of the clinical situation.^46^

To determine whether a UTI is complicated, which subgroup a child belongs to, and which investigations are needed, elements of the medical history and clinical review should include:

- primary (first) or secondary (recurring) infection, including type (febrile or non-febrile UTIs)
- abnormalities of the urinary tract [on pre- or postnatal ultrasound scan (USS)]
- previous medical/surgical history
- voiding habits
- bowel habits, including history of constipation
- family history or nephrological/urological problems
- neonates and infants with urosepsis may present with nonspecific symptoms (fever without source, failure to thrive, jaundice, vomiting, hyperexcitability, lethargy, hypothermia and sometimes without fever)
- in older children upper urinary tract symptoms include fever, flank pain and vomiting
- in older children, lower urinary tract symptoms include dysuria, frequency, urgency, incontinence, hematuria and suprapubic pain
- malodorous urine may be a symptom of UTI, but it is nonspecific as other factors such as diet, hydration and medication may also cause this.

Additional blood tests^47,48^ and imaging can be useful depending on the subgroup of cUTI, both for initial diagnosis and subsequently (Table 2), with the rationale for their use often different at different stages in the infection. For example, USS of the kidneys and urinary tract during the acute phase can neither rule in nor rule out an upper UTI (pyelonephritis) or vesicoureteric reflux (VUR). Therefore, the role of acute USS in children with urosepsis, septic shock, poor urine flow, abdominal or bladder mass and increased creatinine is to determine whether there is acute obstruction that needs urgent surgical intervention.^49^ USS may be performed for different reasons, including failure to respond with suitable antibiotics within 48 hours to investigate for renal abscess, or for recurrent UTI to assess for other urological abnormalities needing intervention.

All children should be reassessed 48 hours after diagnosis for (1) clinical response to treatment, (2) confirmation of the diagnosis and (3) potential targeting of antibiotics according to susceptibility (aim to the narrow antimicrobial spectrum).^29,50^

Treatment should be stopped if the UTI diagnosis is not confirmed, that is, most cases with a negative urine culture with no prior antibiotics to explain this. If the response to treatment is atypical and does not follow the expected trajectory after 48 hours, reassessment for the cause is imperative, rather than simply switching to IV or broader-spectrum antibiotics (although either of these could be needed). Depending on the clinical situation, repeat urine culture, inflammatory markers, serum creatinine and renal USS may be needed to confirm the UTI diagnosis and assess for complications.^51^ Other investigations may be needed to seek an alternate/additional diagnosis.

### How Should Children With cUTI be Treated?

Treatment of cUTI involves consideration of antibiotic route, choice and duration and supportive management. The majority of RCTs and meta-analyses of childhood UTIs exclude those with cUTIs, so recommendations largely rely on a synthesis of retrospective studies (see Table, Supplemental Digital Content 2, http://links.lww.com/INF/G141) and expert opinion to provide a practical empirical guideline to initiate treatment (Table 3). Due to the variability of complicating features even within subgroups, a further individualized approach is often needed, considering local antibiograms, past urine cultures, antibiotic exposure, hospital admissions, urine catheterization and other host factors, including allergies.

**TABLE 3.** Antibiotic Treatment for Subgroups With cUTI, Including Route, Choice and Duration

cUTI Subgroup	Route	Empirical Choice Examples/Classes	Duration (d)	Evidence Grade
Known significant urological abnormality*				
Obstruction or VUR grades 4 and 5	Initial IV then oral	Aminoglycoside OR broad-spectrum B-lactam	IV: Until afebrile/well Total: 10–14	B–D
VUR grades 1–3, other	Oral	Narrow-spectrum B-lactam	Total: 10	C–D
Multiple recurrent UTI†				
Known past resistance	IV if no oral option, else oral	Use previous susceptibility: eg, 2nd line aminoglycoside or fosfomycin	Total: 7–10	C–D
High risk of resistance	Oral	TMP/SMX or quinolone	Total: 7–10	B–D
Low risk of resistance	Oral	Narrow-spectrum B-lactam	Total: 7–10	B–D
Severe clinical presentation				
Sepsis (tachycardia when afebrile, low BP prolonged CRT and severe dehydration)	Initial IV then oral	Broad-spectrum B-lactam	IV: Until afebrile/well Total: 10–14	C–D
Extensive renal parenchymal disease (nephronia and abscess)‡	Initial IV then oral	Broad-spectrum B-lactam OR aminoglycoside	IV: Until afebrile/well Total: 14–21	B–C
Nonurological underlying conditions				
Postrenal transplant	Initial IV then oral	Broad-spectrum B-lactam	IV: Until afebrile/well Total: 14	D
Other renal impairment	Oral	Narrow-spectrum B-lactam or TMP/SMX	Total: 10	D
Nonrenal: immunocompromised	IV if FN, else oral	IV: B-lactam/B-lactamase or anti-pseudomonal B-lactam; Oral: broad-spectrum B-lactam or TMP/SMX	IV: Until afebrile/well Total: 10	B–D
Other	Oral	Narrow-spectrum B-lactam	Total: 7–10	C–D
Neonates and infants <2 m				
With bacteremia	Initial IV then oral	Aminoglycoside ± narrow-spectrum penicillin	IV: ≤7 Total: 10–14	C–D
No bacteremia	Initial IV then oral	Aminoglycoside ± narrow-spectrum penicillin	IV: ≤3 Total: 10–14	C–D

B-lactam indicates beta-lactam antibiotic; BP, blood pressure; CRT, capillary refill time; FN, febrile neutropenia; TMP/SMX, trimethoprim/sulfamethoxazole.

* Including postsurgery/instrumentation; if urological abnormality has led to recurrent UTIs, consider broader empiric choice.

† If other cUTI group (eg, urological abnormality or severe presentation) follow appropriate recommendations.

‡ Aminoglycosides concentrate well in the urine but have less penetration into renal parenchyma due to their hydrophilic nature, so choice depends on pathogen and initial progress.

### Initial Antibiotic Route

- For most children with cUTI, oral antibiotics can be trialed initially, including those who are not severely unwell, for isolated urological abnormality (including post-surgery or urological implementation), isolated tachycardia and for most underlying conditions (GRADES C and D).
- The exceptions for whom at least initial IV is recommended are significant urological abnormality (VUR grades 4 and 5 or obstruction), clinical features of sepsis, renal nephronia/abscess, neonates and infants <2 months, post-renal transplant and immunocompromise with concomitant febrile neutropenia (GRADES B–D).

The primary rationale for administering IV antibiotics is to rapidly achieve high concentrations at the site of action in critical situations while bypassing the limitations of oral intake, absorption and bioavailability. There are few studies including children with significant anatomical and functional urologic abnormalities. The only high-grade evidence for route of antibiotic for VUR is from an RCT that included isolated VUR.^52^ For VUR grades 1 and 2, oral antibiotics were as effective as IV. For VUR grades 3–5, it appeared that there was less scarring if IV antibiotics were used: 8/24 (33%) scarring with oral versus 1/22 (5%) with IV, *P* = 0.02. However, only 16% of participants had VUR grades 3–5 so the study was underpowered for this group and the authors did not draw conclusions. There are no other IV versus oral studies for VUR grades 3–5. A prospective study found that half of 172 children with any urological abnormality were treated with oral antibiotics with good clinical outcomes, although this was not stratified by severity.^53,54^ Given the move towards oral antibiotic management over the last 2 decades since the above RCT, and assessing clinical outcomes rather than dimercaptosuccinic acid (DMSA) scans, we recommend that most children with VUR grade 3 can be managed with oral antibiotics. There are no studies comparing oral with IV post instrumentation or surgery in this group, but in a stable child, oral antibiotics are appropriate.

For children with multiple recurrent UTI, starting with IV antibiotics is not obligatory. Each episode should be managed according to presentation. This group is at risk of infection with antibiotic-resistant bacteria through increased antibiotic exposure.^55,56^ If a child has previously had a UTI with bacteria resistant to all oral options, initial antibiotics can be IV until culture results are available. Intravesical antibiotics, via the urethra or Mitrofanoff, are occasionally used to treat intractable recurrent UTI, with small studies showing successful outcomes with 7 days of treatment.^57,58^ Local irrigation may limit systemic antibiotic side effects and keep children out of hospital.

UTI with fever alone can be effectively treated with oral antibiotics.^59^ Severe clinical presentations of UTI requiring initial IV include sepsis (features include hypotension and tachycardia when afebrile) and extensive parenchymal involvement.^5,27,29,34,60^ Vomiting can be a sign of sepsis, but on its own is rarely a reason to start IV: in an observational study, over 60% of children with vomiting were successfully treated with oral antibiotics.^53,54^ For extensive renal parenchymal involvement (nephronia/abscess), IV antibiotics ensure maximum concentration at the site.^61^

Nonurologic underlying conditions are renal and nonrenal. A review of UTI postrenal transplant recommended initial IV antibiotics for pyelonephritis to achieve rapid tissue saturation.^62^ Although there is a lack of studies of UTI management with other renal impairment, in the absence of severe VUR, oral antibiotics can usually be used. For immunocompromised children, treatment should be via febrile neutropenia pathways if relevant. Otherwise, unless presenting severely unwell, oral antibiotics may be appropriate in immunosuppression or other states of chronic disease (eg, diabetes and liver disease).

For neonates and infants <2 months, there are no RCTs and little comparative evidence to definitively guide the route of antibiotics for UTI. Major guidelines recommend IV antibiotics due to suboptimal enteral absorption, immune immaturity and high risk of bacteremia.^5,27,29,34,60^ The only study of oral antibiotics in infants that included this age group had too few <2 months to make strong recommendations supporting this.^63^ Most retrospective studies of UTI in neonates^64^ and infants <2 months^65–71^ used initial IV antibiotics, and until there is any evidence to the contrary, this continues to be the recommendation, at least as the initial route.

Several studies support home IV treatment through well-resourced hospital-in-the-home programs if children are stable,^3,72–74^ including neonates.^75^ In deciding between initial IV or oral antibiotics, the concept of complicated UTI being on a spectrum may be useful. Apart from children with sepsis, the majority with a single complicating feature are initially managed orally.^53,54^ Increasing the number of complicating features increases IV use, so they could be combined into a clinical score with a threshold for using IV.^76^ Outside of severe presentation, severe uropathy and neonates, clinically stable children with isolated complicating features can initially be treated with oral antibiotics.^34^

### Empiric Antibiotic Choice

- Regarding empiric antibiotic choice (1) different cUTI subgroups have different uropathogen prevalence and (2) an individualized risk assessment for resistance is needed based on the child’s underlying condition, prior antibiotic use and hospitalization. Based on these, an empiric regimen can be chosen that covers likely pathogens and incorporates the likelihood of resistance (GRADES C and D).
- Patients with recurrent infections may initially be treated based on previous cultures (GRADE D).
- For children on antibiotic prophylaxis, a different antibiotic choice is needed (GRADE C).
- Targeted de-escalated monotherapy is recommended according to susceptibilities once available (GRADE D).

Each cUTI subgroup is vulnerable to different pathogens, and assessment for unusual and multidrug-resistant (MDR) bacteria is crucial.^77^ Risk factors for ESBL-producing Enterobacterales (ESBL-E) include recurrent UTI, VUR, recent antibiotic exposure, young age and *Klebsiella* spp.^78–80^ Risk factors for more resistant carbapenemase-producing uropathogens are prolonged hospitalization, invasive devices and recent travel to endemic areas.^78–80^ Empiric antibiotic choice needs to balance the risk of resistance with the need for antimicrobial stewardship for this common infection, potentially accepting a short delay in definitive treatment for children who are hemodynamically stable.

Common uropathogens in children with significant urologic abnormalities, including postsurgery and instrumentation, are Enterobacterales, *P. aeruginosa*, *Enterococcus* spp. and *Staphylococcus aureus*.^78,81–85^ To cover these, if IV antibiotics are needed, an aminoglycoside or broad-spectrum cephalosporin is recommended, with a child’s individual history guiding whether to cover rarer *S. aureus* or more indolent *Enterococcus* spp. empirically.^80,83^ Where multiple antibiotics have previously been used, assessment of the likelihood of resistance is part of management (see below).

Multiple recurrent UTIs, defined by 2 or more UTIs caused by different organisms,^86^ are a risk for resistant and MDR bacteria.^83^ Although recurrent UTIs might occur without anomalies of the kidneys and urinary tract, other risk factors include VUR, underlying neurological conditions, prolonged hospitalization and UTI prophylaxis, and girls are at higher risk than boys.^84,87,88^ If on prophylaxis, a different treatment antibiotic is required due to breakthrough. For children at high risk of resistance, including suspected ESBL-E, empiric oral options include nitrofurantoin, trimethoprim-sulfamethoxazole, quinolones and fosfomycin.^89^ Empirical IV options for children at moderate risk of resistance but who have never cultured ESBL-E include gentamicin and quinolones. If there has been previous ESBL-E or clinical risk is higher, IV amikacin retains high susceptibility to uropathogens, concentrates well in urine and can avoid broad-spectrum beta-lactam and carbapenem use.^59,90^ Most gentamicin-resistant Enterobacteriaceae exhibit cross-resistance to tobramycin but remain susceptible to amikacin, due to reduced inactivation by bacterial enzymes.^91^ If cultures grow *Klebsiella pneumoniae* with high bacterial inoculum, empirical B-lactam antibiotics should be switched to a different class. With a high bacterial inoculum, the stationary phase is rapidly reached, so the effect of targeting penicillin-binding proteins is rapidly diminished, and an alternative is better.^92^ In the large majority, carbapenems should be spared since their use is the greatest risk for developing carbapenem resistance. Even with a history of recurrent UTI with resistant uropathogens, empirical carbapenems are rarely needed in children with cUTI unless a child presents with sepsis (see below).^93–95^ If the identified uropathogen is MDR, including carbapenem-resistant, options include a carbapenem plus amikacin combination, colistin and newer B-lactam/B-lactamase antibiotics such as ceftazidime-avibactam.^80,93^ Since methicillin-resistant *S. aureus* (MRSA) is an uncommon uropathogen, this rarely needs to be covered empirically. *P. aeruginosa* (susceptible and resistant) is more common in recurrent UTIs, so it should be empirically treated with oral quinolones or IV aminoglycosides.^83^

For children with severe clinical presentation with sepsis features and no previous risk factors for resistance, *E. coli* is the most common pathogen, followed by *Klebsiella*, *Enterococcus*, *Proteus* and *Pseudomonas* spp.^60,96,97^ Treatment with broad-spectrum beta-lactam antibiotics such as third-generation cephalosporins is recommended in moderately unwell children. If a child is severely unwell with sepsis potentially of urinary origin, local sepsis guidelines should be followed, considering the individual child’s risk and/or history of resistance and whether it is hospital-acquired. There is occasionally a case for using empirical carbapenems in children with cUTI, for example, in a child with sepsis and a history of highly resistant organisms. This should be in consultation with an infectious diseases specialist to tailor empirical treatment and switch, when possible, with susceptibility.

For nephronia/renal abscess, *E. coli* is the most common pathogen, but urine culture is frequently negative.^61^ Since urine culture may not identify the organism or its susceptibility, if progress is poor, penicillin/ampicillin may be added to cover *Enterococcus* spp. Early consultation with infectious diseases and urology is also recommended in case abscess drainage is needed. In specific clinical circumstances, *S. aureus* (including MRSA), *C. albicans* and *Mycobacterium tuberculosis* may cause renal abscess.^98,99^

Children with nonurologic underlying conditions have different risks of unusual organisms and resistance depending on hospitalization and antibiotic exposure. In children postrenal transplant, especially with urological abnormalities, there is a greater likelihood of *Enterococcus* spp., *S. aureus* or *P. aeruginosa.*^62,100,101^ In immunocompromised patients, in addition to Enterobacterales and *P. aeruginosa*, less virulent organisms such as CoNS, *Haemophilus influenzae* and group B streptococcus may cause cUTI.^62,100,101^ Fungal infections and viruses, for example, adenovirus, can also infect the urinary tract.^101,102^ Choice of antibiotic in chronic renal impairment, immunocompromise or other chronic disease (eg, diabetes mellitus), therefore, depends on likely pathogens and risk of antibiotic resistance based on past antibiotic use and hospitalization. Mild acute or chronic renal impairment does not preclude use of aminoglycosides, which are generally well tolerated in children with renal dosing adjustment. Blood levels should be monitored closely. In moderate/severe renal failure, aminoglycosides should be used only when there is no alternative, as there is a higher risk of accumulation and an unknown risk to hearing.^103^

In neonates and infants <2 months, common pathogens include *E. coli*, *Klebsiella* spp., *Proteus* spp., *Enterobacter* spp., group B streptococcus and *Enterococcus* spp.^104,105^ In preterm infants, hematogenous transmission is more prevalent, with CoNS and *Candida* spp. being causative pathogens.^105^ Empirical IV ampicillin and gentamicin are recommended for neonatal UTI by the World Health Organization.^106^ Resistance should be considered with maternal history of antibiotics during pregnancy^107^ or maternal ESBL-E cultured.^108^ Empirical treatment should be based on maternal isolates, although if unable to access these, gentamicin is usually sufficient. For prolonged stays in neonatal intensive care, resistant hospital pathogens, including MRSA,^109^ should be considered with empirical vancomycin if the infant is severely unwell.

Once culture results are available, empiric antibiotics can be targeted and potentially narrowed.

### Antibiotic Duration and Timing of IV to Oral Switch

- For most children with cUTI, there is not enough evidence to support varying from current practice and expert opinion recommending a total of 10–14 days (GRADE D).
- When IV antibiotics are started, recommended IV durations vary between ≤3 days and ≤7 days depending on the cUTI subgroup and presence of bacteremia, with an early switch when the child is afebrile and clinically well (GRADES C and D).
- Longer total durations (up to 21 days) may be needed for renal abscesses or nephronia, and renal USS is recommended after 14 days to determine progress and ultimate antibiotic duration (GRADE B).
- For frequently relapsing UTI with the same organism, investigations should assess for an ongoing focal source of infection (GRADE D).
- Shorter total durations (6–9 days) and a single dose IV component show early promise, but prospective studies are needed with more children with complicated UTI (GRADES C and D).

Treatment durations for lower and upper UTIs have gradually reduced, but randomized controlled trials and meta-analyses of shorter durations have largely excluded children with complications.^59,110,111^ Therefore, there is scant evidence for total durations shorter than 10–14 days for cUTI.

For neonates and infants <2 months, the majority of retrospective studies in bacteremic^65,67,68,112^ and nonbacteremic^64,67,68,70,71,113–116^ UTI used a total of 10–14 days of antibiotics with high success rates. A recent systematic review comparing the IV component in infants up to 3 months recommended: for bacteremic UTI, IV antibiotics up to 7 days; and for nonbacteremic UTI, up to 3 days.^117^ Switching from IV to oral in both situations is recommended when the infant is afebrile, well and tolerating oral intake.

For severe clinical presentations, a retrospective study of children with febrile UTI included those with initial tachycardia, hypotension and/or vomiting.^118^ It showed that 6–9 days was as effective as ≥10 days of total antibiotics, although it did not report numbers with severe features. Once features of sepsis resolve (usually by day 3),^119^ total antibiotic duration is dependent on bacteremia. A multicenter study of children under 6 months with UTI found bacteremia was more likely to be treated with ≥4 than ≤3 days IV (total 7–14 days).^120^ A case-control study comparing bacteremic versus nonbacteremic UTI found a difference in IV (5–7 vs. 3–4 days) but not total (11–12 days) antibiotic duration, and no differences in outcomes.^116^ For acute lobar nephronia, a review including overlapping RCTs compared 14 versus 21 days’ total duration, with initial IV then switching to oral 2–3 days after defervescence.^61,121^ There was higher treatment failure with 14 days, but failure was associated with longer pre-presentation fever, suggesting longer treatment is necessary for more established abscesses.

For nonurologic underlying conditions affecting the kidneys, RCTs that did not exclude renal impairment showed no difference comparing ≤3 versus ≥4 days IV of a total of 8–15 days, although renal impairment was not analyzed separately.^117,122^ Postrenal transplant, expert opinion recommends 14 days total for febrile UTI/pyelonephritis, switching from IV with clinical improvement.^62^

Although immunocompromised children were unusually included in a study of febrile UTI, numbers were too small to extrapolate findings of shorter antibiotic durations.^118^ A small study found children with cancer often have few classic UTI symptoms,^123^ so diagnosis and definitive treatment may be delayed. Immunocompromised children usually have multiple admissions and antibiotic courses, so they are more likely to have unusual bacteria or resistance. Once definitive treatment starts, there is no reason for prolonged courses in this or other chronic conditions (eg, diabetes and liver disease).

For children with significant urologic abnormalities, the only study comparing total antibiotic duration included 23% with urological abnormalities and found no difference between 6–9 and ≥10 days in treatment failure/recurrence, although it did not analyze them separately.^118^ Two RCTs investigating IV duration for febrile UTI included 34% and 37% children with VUR, respectively, and found no difference between 3 and ≥7 days IV of a total 8–15 days.^117,122,124^ A retrospective study including 1930 children with urological abnormalities found they were more likely to be treated with ≥4 days than ≤3 days IV (total 7–14 days), but this was not associated with outcomes.^120^ A retrospective study including urological abnormality compared 1 day versus 2–3 days’ IV (median total 10 days) and found no difference in readmission, although outcomes were not analyzed by urological abnormality.^32^ Recruitment is currently underway for an RCT of cUTI comparing 1 versus 3 days of IV of a total of 7 days of antibiotics.^125^

For children with multiple recurrent UTIs, antibiotic duration should be tailored to each individual acute UTI presentation. Multiple antibiotic courses for recurrent UTIs increase the likelihood of resistance.^84^ Longer admissions (although not antibiotic durations) have been shown for ESBL-E UTIs than for non-ESBL-E UTI.^84,126,127^ IV treatment via hospital-in-the-home programs could shorten hospital length of stay.^3^ For resistant UTIs, a comparison of IV durations of ≥3 days with 0–2 days showed no difference in recurrence, although total duration was not reported.^128^ Definitive antibiotic treatment should not usually need a longer duration simply due to resistance.

### Supportive Management

- Additional management depends on the cUTI subgroup and complicating features. This may involve antiemetics, fluid resuscitation and management and occasionally intensive care support. There is no clear role for steroids currently (GRADE D).
- Surgically, temporary diversion may be needed for urinary obstruction, and percutaneous drainage is considered for renal abscesses (GRADE D).

Modes of supportive care for cUTI are dependent on subgroup and presenting features. Severely ill patients may need fluid resuscitation and intensive care support. Patients with vomiting should have antiemetics, as this may avoid use of IV antibiotics. With obstructive uropathy, temporary urinary diversion may be needed if antibiotics alone fails.^5^ For renal abscesses >3 cm, percutaneous drainage should be considered.^17,61,98,99^ A recent meta-analysis concluded that steroids reduced renal scarring associate with UTI.^129^ However, the included studies either did not show this at all,^130^ or did not reach statistical significance. Given the variations between included studies in inclusion criteria, intervention and timing and the uncertain clinical impact of scar reduction, there is no clear role for steroids in UTI currently. No other medications have been identified as beneficial.

### How Should We Follow Up Children With cUTI?

Follow-up of children with cUTI involves consideration of antimicrobial prophylaxis, imaging and referral to nephrology/urology.

### Antimicrobial prophylaxis

- The benefit of continuous antibiotic prophylaxis is controversial and reserved for particular cUTI subgroups: (1) in neonates and infants <2 m, this includes those with high-grade VUR (3–4), antenatal hydronephrosis (HN), ureteral dilatation and HN in uncircumcised infants; and (2) in older children, this includes those with multiple recurrent UTI, high-grade VUR (3–4), spina bifida accompanied with high-grade VUR and/or intermittent catheterization, bladder/bowel dysfunction and severe obstructive uropathy until surgical correction (GRADES B–D).
- In neonates, amoxicillin is the drug of choice, while older children are recommended to use trimethoprim-sulfamethoxazole or nitrofurantoin (GRADES C and D).
- Consider use of cranberry products in older children to alleviate antibiotic pressure (GRADE B).
- Duration of CAP should not exceed 3–6 months followed by critical reevaluation (GRADE D).

### Effectiveness for cUTI Subgroups

Antimicrobial prophylaxis aims to prevent UTI recurrence. Several RCTs have shown that after 1 UTI, even with VUR grades 1 and 2, there is no benefit to continuous antimicrobial prophylaxis (CAP), so it is not routinely recommended.^27,29,60,131–133^ However, studies have shown benefits for some subgroups^134^ with cUTI (Table 4).

**TABLE 4.** Follow-Up of cUTI Subgroups With Continuous Antimicrobial Prophylaxis and Imaging

cUTI Subgroup	Recommendation for Continuous Antibiotic Prophylaxis	Follow-Up Imaging	Evidence Level
Significant anatomical or functional urological abnormality	• Spina bifida with high-grade VUR and/or CIC • High-grade VUR (3 and 4) • Bladder/bowel dysfunction • Severe obstructive uropathy until surgical correction	Repeat USS Consider repeating MCUG and DMSA Uroflowmetry for bladder/bowel dysfunction	A–B
Multiple recurrent UTI	>3 UTI in 12 mo	USS Consider repeating MCUG/DMSA Consider cystoscopy	C
Severe clinical presentation	Not routinely (if first episode)*	USS Consider further imaging with MRI/CT/DMSA	D
Nonurological underlying conditions (eg, renal transplant)	Not routinely (if first episode)*	Repeat USS Consider further imaging with MRI/CT/DMSA	C
Neonates	• High-grade VUR (3 and 4) • Antenatal hydronephrosis (HN) • Ureteric dilatation and HN • HN and no circumcision	USS Further imaging according to pathology	C

* Consider if ≥2 episodes, in consultation with pediatric infectious diseases and pediatric nephrology specialists.

For children with known significant urologic abnormalities, the most well-studied has been VUR. Several multicenter RCTs have shown significant reduction in UTI recurrence in children with VUR grades 3–5, although without reducing scarring.^88,135,136^ In 1 RCT, the impact was particularly high in children with bladder-bowel dysfunction.^136^ In a prospective study of myelomeningocele, infants with VUR grades 3 and 4 or clean intermittent catheterization had increased UTI risk, so the authors suggested CAP for these 2 situations.^137^ CAP may be beneficial in children with posterior urethral valve obstruction until surgical correction, but evidence is lacking. Lack of evidence further hampers recommendations regarding the ongoing duration of prophylaxis after ureteral implantation, ablation of posterior urethral valves or after endoscopic treatment of VUR.^138^

Multiple recurrent UTI are common—over one-third of children with UTI after their first birthday experience recurrence.^139^ Preventing recurrent UTI may prevent renal scarring, and although there remains debate about the contribution of UTI to scarring,^139,140^ the latter can lead to hypertension and renal insufficiency later.^28^ Prevention also avoids pain, potential sepsis, hospital admission and family disruption. Risk factors for multiple recurrent UTI are high-grade VUR, bladder-bowel dysfunction and the combination of both.^141^ A meta-analysis showed that the number of children receiving CAP needed to prevent one recurrent UTI was 21.^142^ A Cochrane review showed that although there was a modest reduction in recurrence while on CAP—both with VUR and without—the confidence intervals were wide and concluded that any small benefit gained should be weighed against the risk of inducing antibiotic resistance.^143^

Children with severe clinical presentation need further investigation regarding urological pathology and VUR. In the absence of these, there is no evidence of benefit for CAP in children after severe presentation.

The most relevant non-urologic underlying condition is renal transplant. Although acute UTI negatively impacts graft function, it remains unclear whether CAP can prevent posttransplant UTI.^144,145^ Children with other conditions may receive antibiotic prophylaxis (eg, immunocompromised), but there is no data supporting the benefit specifically to prevent UTI.

Although there are no RCTs in neonates and infants <2 m, systematic reviews suggest CAP may be useful in high-grade antenatal hydronephrosis, hydronephrosis with ureteral dilatation or hydronephrosis in uncircumcised infants.^146^

### Antibiotic Prophylaxis Choice, Duration and Resistance

The most common agents used for CAP are amoxicillin in neonates and trimethoprim-sulfamethoxazole (TMP-SMX) and nitrofurantoin in older children.^28,29^ Amoxicillin-clavulanate and cephalosporins lack data regarding efficacy and the development of antimicrobial resistance.^138^ Two RCTs showed that 6 months of nitrofurantoin was more effective at reducing recurrence than TMP-SMX, although nitrofurantoin resulted in more gastrointestinal side effects.^147,148^ Although both nitrofurantoin and TMP-SMX are generally considered safe for long-term prophylaxis, nitrofurantoin can also cause cutaneous reactions,^149^ while TMP-SMX can cause nausea and loss of appetite, and more rarely bone marrow suppression, nephrotoxicity and Stevens-Johnson syndrome.^150^ It is therefore recommended not to use TMP-SMX in some hematologic disorders, to dose adjust in renal failure and to monitor full blood count and electrolytes while taking it.^151,152^ The benefits of nitrofurantoin, in terms of efficacy, are counterbalanced by the side effects of the drug, so overall there is little to choose between recommending nitrofurantoin or TMP-SMX for prophylaxis.^143,149,153,154^ A recent meta-analysis confirmed that nitrofurantoin had the greatest likelihood of antibiotic choices in reducing UTI in children but also showed daily cranberry products reduce symptomatic UTI by 59%, more than either TMP-SMX or trimethoprim.^155^ This finding included children with recurrent UTI and VUR. Given that all antibiotic use is associated to some degree with the development of antibiotic resistance, cranberry products offer a promising alternative.

There is a lack of evidence for prophylaxis duration or criteria for discontinuation. Pooled results from 3 observational studies showed that continuation of CAP did not prevent recurrent UTI after pyeloplasty, so this should be a reason to stop.^138^ There is insufficient evidence regarding the duration of CAP after ureteral reimplantation or endoscopic treatment of VUR.^138^ Most commonly, 3–6 months are recommended, followed by reassessment.^156^

Multiple RCTs clearly show that while UTI is less common with prophylaxis, resistance to the prophylactic antibiotic is significantly higher.^56,131,157^ With resistance to either TMP-SMX or nitrofurantoin, switching to the other can be considered, although there is a lack of evidence to recommend a switch after a breakthrough UTI. In case of resistance to both agents, one might consider a CAP disruption, as broader-spectrum agents may lead to UTI with increasingly resistant organisms to remaining oral alternatives.

### Further Imaging

- In post-acute infection, urinary tract USS may help define urological abnormalities (GRADE C).
- USS/CT/MRI can be useful to follow a renal abscess (GRADES C and D).
- Micturating cystourethrogram (MCUG) and DMSA are now saved for specific instances where less invasive investigations do not give enough prognostic information, usually for children with severe urological abnormalities (GRADE D).

### Ultrasound Scan

After the first episode of upper UTI, children under the age of 3 years should have a renal tract USS after the acute phase to detect potential structural abnormalities or parenchymal lesions.^60^ Obtaining an USS and MCUG after the first UTI in young children is, however, not strongly supported by high-grade evidence. A prospective study involving 309 children showed that the identified abnormalities, which were found in 12 percent (37/309) of the children, and obtained within 72 hours after diagnosis, did not modify management.^49^ Some guidelines recommend USS in case of atypical or recurrent UTI.^158^ Children who are older than 2–24 months who experience recurrent febrile UTIs should also be evaluated, though USS has a poor sensitivity for detecting mild-moderate VUR. Due to its rarity, there is a gap of evidence regarding recommendations of follow-up in children with suspected nephronia or abscess, but they may benefit from further imaging with MRI or CT.^159^

### Micturating Cystourethrography

Many guidelines recommend performing a MCUG after abnormal USS findings, non-*E. coli* causative pathogens or recurrent UTI.^160^ Combinations of unusual pathogens and pathological USS increase the probability of high-grade VUR to over 50%.^160^

### DMSA Scan

Although the specificity for detecting high-grade VUR after DMSA renal scan is low,^161^ they can detect renal scars, differences in relative renal function between the kidneys and deterioration of renal function or new scars over time.^162^ DMSA and its timing remain controversial for the evaluation of a child after cUTI, but children with high-grade VUR or recurrent UTI should undergo a DMSA scan to keep track of renal damage.^163^ DMSA should be performed 3–6 months after the acute episode so that transient inflammatory changes have resolved.^164^ In 1 study, the incidence of renal scarring was associated with the severity of renal parenchymal infection, with 22% of children with pyelonephritis having scars, 44% with nephronia and 72% with abscess.^165^

### Magnetic Resonance Urography

Magnetic resonance urography is a radiation-sparing imaging technique that provides both anatomic detail and functional information and should be considered in children with known and complex urological conditions.^166^

### Referral

- When there is an ongoing risk of renal damage that is unlikely to improve without surgical intervention, referral to a pediatric nephro/urology team is important. This is most common for children with significant urological abnormalities, including high-grade VUR (GRADE D).

Children with significant urological abnormalities, including but not limited to ureteric obstruction, duplex kidneys with VUR, grades 3–5 VUR, grades 3–4 hydronephrosis and neurogenic bladder, should be discussed acutely with and referred promptly to a pediatric nephro/urology team. High-grade VUR may persist without intervention.^167^ Evaluation of bladder function through urodynamics performed by urologists should be considered with recurrent UTI or with anatomical or functional abnormalities, as the investigation may detect abnormalities such as manageable detrusor overactivity or dysfunctional voiding.^168^

## DISCUSSION AND CONCLUSIONS

Over the last 5 years, new and updated pediatric UTI guidelines from around the world have become increasingly consistent for uncomplicated UTI, but either do not address or differ in definition and recommendations for complicated UTI. This is the first comprehensive guideline focused exclusively on cUTI in children that addresses its diagnosis and management according to the features making it complicated: young age, disease severity/extent and comorbidities. This guideline is comprehensive in addressing gaps in existing guidelines by first highlighting that cUTI is not a uniform entity but rather constitutes a wide range of disease processes. The guideline presents the evidence for diagnosing and managing the subgroups presenting with each of these processes to ensure more individualized patient management. It also reviews emerging evidence for conceptualizing cUTI as an amalgamation of patients’ individual features and attainment of a threshold for more intense management. Regardless of the subgroup, accurate diagnosis is the critical first step in managing children with cUTI for which additional investigations, such as blood culture, serum biochemistry and USS, might be required. While several subgroups benefit from initial IV (sepsis, VUR 3–5, neonates) and broad-spectrum antibiotics (sepsis, high risk of resistance from recurrent UTI and immunocompromise), oral narrow-spectrum antibiotics are recommended for most children with cUTI who are not severely unwell. Prophylaxis is not needed universally in cUTI, and its relatively small benefits in specific subgroups need to be weighed against the risk of resistance, with at least 6-monthly reevaluation for its requirement. Longer-term follow-up should aim to assess the need for surgical intervention to reduce the risk of renal damage.

Despite cUTI being common in children, differences in definition and focus on uncomplicated infection have led to scarce high-grade evidence for management, that is also difficult to synthesize. Apart from RCT-based recommendations for CAP in children with urological abnormality, these guidelines are largely based on retrospective studies and expert consensus in areas where high-quality evidence is currently lacking. In addition, available evidence mostly comes from resource-rich settings, and in resource-limited settings there may be some barriers to accessing some investigations and treatments, for example, nuclear medicine imaging and broad-spectrum antibiotics. However, most first-line investigations in the guideline are broadly available and most antibiotic recommendations are from the World Health Organization Access list which should be available in most countries. Therefore, these guidelines can be effectively implemented in most healthcare settings, even in resource-limited environments.

Future research should include standardized definitions of children with cUTI. Treatment duration and benefits of prophylaxis should be assessed separately for each subgroup, with outcomes not only considering treatment failure but also other factors including antibiotic resistance, side effects and home management in a more holistic way. Furthermore, studies should focus on evaluating the long-term outcomes of children treated according to these guidelines to further validate and refine these recommendations.

## Supplementary Material


