# Supplementary material for: Guidelines for Complicated Urinary Tract Infections in Children: A Review by the European Society for Pediatric Infectious Diseases
Source: Pediatr Infect Dis J. 2025 Mar 19;44(6):e211–23. doi: 10.1097/INF.0000000000004790 (PMC12058373; doi:10.1097/INF.0000000000004790)
Supplement: Supplementary file 1 [file inf-44-e211-s001.pdf]

Supplementary Table 1a: Definitions used in 16 paediatric studies (Suppl. table 1b) of complicated UTI (cUTI)

Complicated UTI (cUTI) studies (n=16)	N (%)
Definition of cUTI	
Clinical presentation and host risk factors	7 (43.7)
Host risk factors only	5 (31.2)
Clinical presentation only	1 (6.0)
Severity grade included	5 (31.2)
Pathogen risk factors included	2 (12.5)
Host risk factors that were included in the definition	
Functional/anatomical urinary tract abnormalities	15 (93.7)
Recurrences	4 (25.0)
Young age (<3 months)	3 (18.7)
Immunosuppression/transplantation	6 (37.5)
Catheterization	4 (25.0)
Antibiotic prophylaxis	1 (6.0)
BBD	1 (6.0)
