# Supplementary material for: Guidelines for Complicated Urinary Tract Infections in Children: A Review by the European Society for Pediatric Infectious Diseases
Source: Pediatr Infect Dis J. 2025 Mar 19;44(6):e211–23. doi: 10.1097/INF.0000000000004790 (PMC12058373; doi:10.1097/INF.0000000000004790)
Supplement: Supplementary file 2 [file inf-44-e211-s002.pdf]

**Supplementary Table 2:** Summary of studies that investigate treatment options for cUTI in children.

| Subtopic | Study | Design | Participants | Objective - key results |
|---|---|---|---|---|
| **Route of administration** | | | | |
| | Autore et al (2022) [32] | Delphi experts' opinion; a questionnaire with 81 questions | Experts' opinion | Management of Pediatric UTIs; infants < 3 mo, critically ill children and those with failure or poor compliance to oral therapy require intravenous therapy |
| | Scanlan et al (2019) [3] | 4-year single-center retrospective observational study | 62 children with pyelonephritis in ED | Treatment of cUTI with outpatient IV antibiotic therapy at home; course successfully completed in 90% of patients |
| **IV-to-oral switch and total duration** | | | | |
| | Lewis-de Los Angeles et al (2017) [61] | 10-year multicenter retrospective observational study | infants <60 days with UTI in 46 children's hospitals | Assessment of trends in IV antibiotics duration; proportion of infants receiving long IV treatment decreased throughout the decade without increase in readmissions |
| | Corrales Magín et al (2007) [59] | Single-center retrospective observational study | 172 neonates (median age 19 days) with UTI | Efficacy of short-term IV antibiotics; no treatment failure or relapse with a median length of 4 days followed by oral treatment |
| | Desai et al (2019) [60] | 6-year multicenter retrospective cohort study | 115 young infants with bacteremic UTI in 11 children's hospitals | Comparison between short vs long courses of parenteral antibiotic; no more frequent recurrent UTIs or hospital reutilization for infants received ≤7 days of IV antibiotics |
| | Strohmeier et al (2014) [55] | Cochrane Database Syst Review to April 2014 | 4452 children 0-18 years with proven UTI and APN | Comparison between different treatment approaches; a short course (2-4 days) of IV therapy followed by oral therapy is as effective as a longer course (7-10 days) of IV therapy. |
| | Keren et al (2002) [150] | Meta-analysis of 16 Randomized Controlled Trials | Children 0-18 years with acute UTI | Comparison between short-course (</=3 days) and long-course (7-14 days) therapy; long-course therapy associated with fewer treatment failures and lower risk of reinfection |
| | Cheng et al (2006) [57] | Single center Randomized Controlled Trial | 80 children with acute lobar nephronia | Comparison of the treatment duration efficacy; a total of 3 weeks of antibiotic therapy should be the treatment of choice |

| | | | | |
|---|---|---|---|---|
| | Fox et al (2020) [109] | 2-year multicenter retrospective observational study | 791 children 6 months - 18 years with pyelonephritis in 5 hospitals | Comparison between short-course (6-9 days) and prolonged-course (>10 days) of antibiotics; short-course may be as effective as prolonged-courses and may mitigate the risk of future drug-resistant UTIs |
| **Pathogens Epidemiolo gy** | | | | |
| | Burckhardt et al (2016) [77] | 5-year single-center retrospective observational study | 18 children & adults with 26 episodes of *S. pneumoniae* UTI | *S. pneumoniae* as an agent of urinary tract infections; higher prevalence in patients post-renal transplant or with other renal dysfunctions |
| | Megged et al (2014) [78] | 13-year single-center retrospective comparative study | 26 children with *S. aureus* UTI (controls: *E. coli* UTIs) | Demographic, clinical, and laboratory data review; *S. aureus* uncommon urinary pathogen with higher rates of urinary abnormalities when detected |
| | Bitsori et al (2012) [79] | 12-year single-center retrospective comparative study | 35 children with 43 episodes of *P. aeruginosa* UTI (controls: *E. coli* UTIs) | Assessment of risk factors for *P. aeruginosa* UTI; to be considered in recent antibiotics exposure with prophylaxis included |
| | Flokas et al (2016) [80] | Systematic review and meta-analysis of 16 studies | 7374 children with ESBL-UTIs (controls: other UTIs) | Assessment of risk factors for ESBL *Enterobacteriaceae* UTI; to be considered in VUR, previous UTI or recent antibiotic use |
| | Vachvanichsanong et al (2020) [74] | 10-year single-center retrospective comparative study | 37 boys and 46 girls with 102 episodes of ESBL-UTI (controls: non-ESBL UTIs) | Investigation of prevalence, clinical findings, impact, and risk factors of ESBL E. coli/K. pneumoniae UTI; represented one-third of E. coli/K. pneumoniae UTI episodes with recurrent UTI being the sole independent risk factor identified |
| | Ahn et al (2022) [75] | 7-year single-center retrospective observational cohort study | 330 adults with ESBL-UTIs | Association between patient characteristics and the development of recurrent UTI caused by ESBL *Enterobacteriaceae;* previous frequent UTI recurrence identified as the sole risk factor |
| | Arshad et al (2015) [97] | Literature review | Neonates and young infants with UTI | Diagnosis & management approaches; *E. coli* the most common pathogen. Premature infants at increased risk for *Candida* spp |
| | Walawender et al (2020) [96] | 5-year single-center retrospective observational study | 28 neonates admitted to NICU in the first | Evaluation of urine culture results versus established UTI criteria. equal distribution of *E. cloacae, E. coli, K. pneumoniae* and coagulase (-) *Staphylococcus* |

| | | | | |
|---|---|---|---|---|
| | | | 3 months with UTI | |
| | Valera et al (2006) [93] | 2-year single-center prospective observational study | 161 adults received kidney transplant, all on prophylaxis with sulfadoxine-pyrimethamine | Epidemiology of UTIs; *E. coli* as the principal isolated agent in 71% of UTI cases, 24% of which ESBL |
| **Antimicrobial resistance & Empiric Choice** | | | | |
| | Mattoo et al (2021) [56] | Literature review | Infants and children with UTI | Contemporary management of UTI in Children; 85-90% of UTIs caused by *E. coli. Klebsiella, Proteus, Enterococcus and Enterobacter* spp also common. Majority of uropathogens sensitive to third generation cephalosporins |
| | John et al (2009) [58] | Literature review & Recommendations | Children with febrile UTIs after renal transplantation | Diagnosis and management approaches; *Enterococcus* and *Pseudomonas* spp more frequent. Recommendation of treatment with ceftazidime and ampicillin |
| | Esposito et al (2021) [89] | 8-Year multicenter retrospective observational study | Patients <18 years hospitalized for UTI in one region | Antibiotic Resistance assessment: amoxicillin/clavulanate no longer appropriate first-line therapy, whereas third generation cephalosporins continue to be helpful |
| | Buschel et al (2022) [90] | 11-year single-center retrospective comparative study and literature review | 14 children with renal abscess | Renal abscesses in children; *S. aureus* to be considered when prescribing empirical antibiotics. Percutaneous drainage to be considered in cases of antibiotic failure. |
| | Zhang et al (2019) [91] | 10-year single-center retrospective observational study | 17 children with renal abscess | Analysis of children with renal abscess; to consider anti-Gram (+) and fungal treatment, the possibility of renal TB infection and surgery in abscesses >4 cm not responsive to drug therapy |
| | Bitsori et al (2019) [76] | Literature review & Recommendations | Children with UTI caused by ESBL *E. coli / K. pneumoniae* | Risk factors & treatment approach; exposure to antibiotics identified among others as the main risk factor. Carbapenems remain the cornerstone of treatment, whilst alternative agents are worth of consideration in less severe cases |

| | | | | |
|---|---|---|---|---|
| | Yildirim et al (2008) [151] | Prospective, randomized clinical trial | Children with acute lymphoblastic leukemia or acute myeloblastic leukemia and febrile neutropenic episodes | Comparison of efficacy between piperacillin/tazobactam and amikacin combination and carbapenem monotherapy as empiric choice of treatment; equal efficacy |
| | Autore et al (2022) [88] | 8-year multicenter retrospective observational study | 1801 children with febrile UTIs in one region | Clinical outcomes of discordant empirical treatment and risk factors associated to treatment failure; it may still be effective in more than half of pediatric UTIs (failure rate 47.2%) |
| | Han et al (2015) [152] | 5-year single-center retrospective comparative study | 205 children with 211 episodes of UTI due to *E. coli* or *K. pneumoniae* | Comparison of clinical characteristics and therapeutic responses between ESBL and non-ESBL group; favorable therapeutic effects of non-carbapenem antibiotics for ESBL-producing strains. Aminoglycosides to be considered as a good alternative |
| | Sethaphanich et al (2016) [84] | single-center retrospective observational study | 849 children with *E. coli* or *K. pneumoniae* infections (40% ESBL positive) | Antibiotic susceptibility of ESBL-producing and non-ESBL-producing strains; amikacin, cefepime and piperacillin/tazobactam are possible options for consolidative therapy or for non-serious infection |
| | Moxon et al (2016) [86] | Literature review | Children with Gram (-) infections | Characteristics of different Beta-lactamases categories (extended spectrum beta-lactamases ESBL, AmpC beta-lactamases and carbapenemase producing Enterobacteriaceae CPE) |
| | Chiotos et al (2016) [877] | Literature review | Children with multi-drug resistant Gram (-) infections | Carbapenem-Resistant Enterobacteriaceae Infections in Children |
| | Baquero-Artigao et al (2019) [83] | Literature review & recommendations | Children with multidrug-resistant infections | Fosfomycin in the pediatric setting |
| **Supportive management** | | | | |
| | T'Hoen et al (2021) [28] | 5-year literature review & recommendations | Children with UTIs | Update of the EAU/ESPU guidelines; percutaneous drainage for abscessed >3cm |

| | | | | |
|---|---|---|---|---|
| | Rius-Gordillo et al (2022) [119] | Multicenter, prospective, double-blind, placebo controlled RCT | 91 children with APN received a 3-day course of either dexamethasone or placebo | Effectiveness of Dexamethasone to prevent kidney scarring in acute pyelonephritis; no effect on reducing the risk |
